# Supramolecular-jack-like guest in ultramicroporous crystal for exceptional thermal expansion behaviour

**DOI:** 10.1038/ncomms7917

**Published:** 2015-04-21

**Authors:** Hao-Long Zhou, Yue-Biao Zhang, Jie-Peng Zhang, Xiao-Ming Chen

**Affiliations:** 1 MOE Key Laboratory of Bioinorganic and Synthetic Chemistry, School of Chemistry and Chemical Engineering, Sun Yat-Sen University, Guangzhou 510275, China; 2 School of Physical Science and Technology, ShanghaiTech University, 100 Haike Road, Shanghai 201210, China

## Abstract

The dynamic behaviours of host frameworks and guest molecules have received much attention for their great relevance with smart materials, but little has been developed to control or understand the host–guest interplay. Here we show that the confined guest can utilize not only molecular static effects but also bulk dynamic properties to control the host dynamics. By virtue of the three-dimensional hinge-like framework and quasi-discrete ultramicropores, a flexible porous coordination polymer exhibits not only drastic guest-modulation effect of the thermal expansion magnitude (up to 422 × 10^−6^ K^−1^) and even the anisotropy but also records positive/negative thermal expansion coefficients of +482/−218 × 10^−6^ K^−1^. Moreover, single-crystal X-ray diffraction analyses demonstrate that the jack-like motion of the guest supramolecular dimers, being analogous to the anisotropic thermal expansion of bulk van der Waals solids, is crucial for changing the flexibility mode and thermal expansion behaviour of the crystal.

## Abstract (web summary)

While host–guest interplay has been well studied, there has been less work on utilizing the bulk property of guest. Here, the authors show that the jack-like motion of guest supramolecular aggregations can drastically modulate the thermal expansion properties of a three-dimensional hinge-like framework.

## Graphical abstract

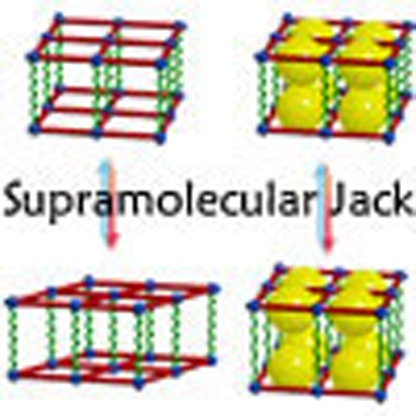

The remarkable framework flexibility is one of the most important advantages of porous coordination polymers (PCPs) or metal–organic frameworks, which allows the host structures to be altered by external stimuli^1^. While chemical stimuli (mainly change of guest) have been extensively used as the driving forces^2^,^3^,^4^,^5^, physical stimuli such as light irradiation and temperature change have been scarcely explored^6^,^7^,^8^,^9^,^10^. Recently, flexible PCPs are emerging as an ideal platform for realizing abnormal temperature-induced structural dynamic behaviours, such as negative thermal expansion (NTE)^11^,^12^,^13^,^14^ and very large thermal expansion (especially for |*α*|>100 × 10^−6^ K^−1^; refs 15, 16, 17, 18, 19, 20), which have great potential for compensation of the small positive thermal expansion (PTE, 0<*α*<20 × 10^−6^ K^−1^) of common solids, designing sensitive thermomechanical actuators, and so on^21^,^22^,^23^,^24^,^25^,^26^,^27^,^28^,^29^. Besides framework flexibility and porosity, the designable framework connectivity of PCP^30^,^31^,^32^ offers an additional approach for engineering the magnitude and anisotropy of thermal expansion^14^,^15^,^16^,^33^,^34^. It should be noted that, for its response in different dimensions, anisotropic thermal expansion is attractive for microdevices and information materials^29^,^35^. More importantly, just like other physical properties^36^,^37^,^38^,^39^, thermal expansion of PCP can be readily guest-dependent, albeit it is still very challenging^16^,^17^,^18^,^19^,^40^,^41^. As the most remarkable examples, Kepert and coworkers achieved isotropic PTE (*α*=+10.0 × 10^−6^ K^−1^) to NTE (*α*=−33.5 × 10^−6^ K^−1^) transition via guest removal in [Cd(CN)_2_]·*x*CCl_4_^41^, and Barbour and coworkers showed that the PTE of [Zn(OH)(niba)]·alcohol (Hniba=4-(1H-naphtho[2,3-d]imidazol-1-yl)benzoic acid) can be increased by ∼90 × 10^−6^ K^−1^ via changing the alcohol guest molecules^18^, in which the microporous structures have been demonstrated to be crucial for realizing the guest-modulation effect, since they maximize host–guest interaction around the guest molecules^17^,^18^,^40^,^41^.

On the other hand, the confinement effect of the porous host allows the formation of low-dimensional guest aggregations with abnormal/appealing physical properties^17^,^39^,^42^. The overwhelmingly large thermal expansion of the fluidic guest (usually beyond 100 times that of common solids) might be utilized to further drive the thermal expansion of the porous host. When the anisotropic structures of the host framework, pore environment and/or the low-dimensional guest aggregation are appropriately coupled, more exciting microscopic/macroscopic phenomenon could be expected. A key to these goals is to visualize, understand and control the delicate host–guest interplay under external stimulus, in which *in situ* single-crystal X-ray diffraction (SCXRD) is the most convenient, straightforward and reliable method^43^,^44^,^45^. Nevertheless, retaining the sample single-crystallinity after guest removal/absorption/exchange and temperature cycling treatments is always a great challenge, and the extremely large mobility of guests also impedes determination of their positions^16^,^17^. Actually, even when the cross-section size of a one-dimensional channel is small enough to fit a single guest molecule well, the guest chain may still show liquid-like mobility^17^. To enable direct observation of thermal expansion of guest aggregations, discrete, isolated or 0D pores with suitable sizes for accommodation of two or a little bit more guest molecules should be optimal^46^,^47^. However, materials with such pores are generally difficult to change guests, preventing rational modulation of the physical property^20^,^48^,^49^.

In this work, we report a PCP possessing not only a three-dimensional (3D) hinge-like structure with multimode flexibility but also quasi-0D ultramicropores suitable for adsorption and confinement of pairs of different amide-type guests. SCXRD studies of this material under temperature- and guest-stimuli reveal the largest NTE and PTE among crystalline solids to the best of our knowledge. We also report a drastic guest-modulation effect, and even a change of the main thermal expansion direction since the steric hindrance and thermal expansion effects of the guest dimers are large enough to alter the flexibility mode of the host framework (Fig. 1).

## Results

### Synthesis and structure of the porous crystal

**Figure 2:**
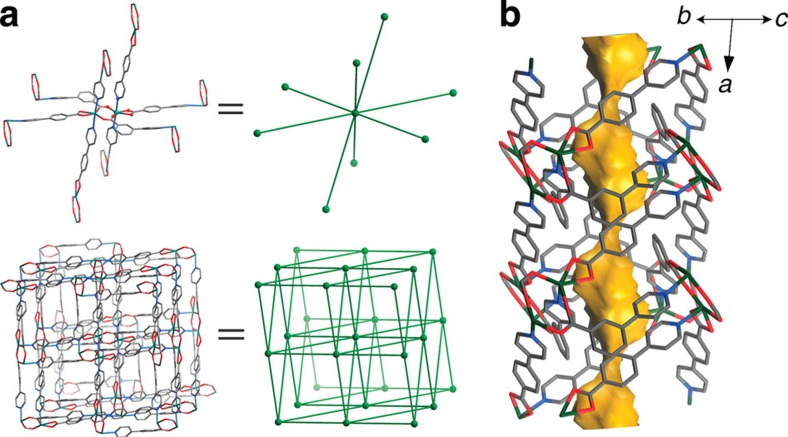
X-ray single-crystal structure of the ultramicroporous framework. (**a**) Perspective view of 3D coordination framework of **1** along the *a*-axis (the aromatic rings of the 34pba^−^ and 44pba^−^ ligands are highlighted in blue and yellow, respectively). (**b**) Solvent-accessible pore surface structure of **1** viewed along the [011] direction, in which large cavities (6.0 × 7.0 × 9.7 Å^3^) are interconnected by very narrow necks (*d*≈1.7 Å).

Solvothermal reaction of Cd(NO_3_)_2_, 3-(pyridin-4-yl)benzoic acid (H34pba) and 4-(pyridin-4-yl)benzoic acid (H44pba) in mixed solvent ethanol/water at 90 °C afforded colourless needle-shaped crystals [Cd(34pba)(44pba)] (**1**, MCF-82). SCXRD analysis showed that **1** crystallizes in the monoclinic space group *P*2_1_/*c* (Fig. 2, Supplementary Fig. 1, Supplementary Table 1 and Supplementary Data 1), containing one Cd^2+^ cation, one 34pba^−^ ligand and one 44pba^−^ ligand in its asymmetric unit. A pair of Cd^2+^ ions are bridged by two exobidentate carboxylate groups from two 34pba^−^ ligands and further coordinated by two chelating carboxylate groups from two 44pba^−^ ligands and four monodentate pyridyl groups from two 34pba^−^ and two 44pba^−^ ligands to form a dinuclear Cd_2_(RCOO)_4_(L_py_)_4_ (L_py_=pyridyl group) unit, which interconnects with eight neighbours through four 34pba^−^ and four 44pba^−^ ligands to form a 3D coordination network with a uninodal 8-connected **bcu** topology (Fig. 2 and Supplementary Fig. 2)^50^,^51^,^52^. Due to the presence of long bridging ligands, the crystal contains a quasi-0D pore system, in which olive-like cavities with cross-sectional size of 6.0 × 7.0 × 9.7 Å^3^ are interconnected through narrow necks (smaller than the diameter of a hydrogen atom) with diameter of 1.7 Å along the *a*-axis.

### Thermal expansion of the porous crystal

**Figure 3:**
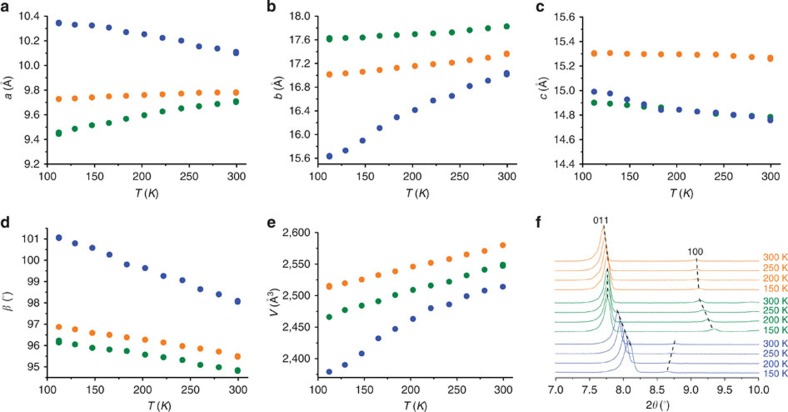
Guest-dependent and guest-induced crystal deformation. (**a**–**e**) Temperature-dependent unit-cell parameters of **1** (blue), **1**·DMF (green) and **1**·DMA (orange) determined by SCXRD. (**f**) PXRD patterns of **1** (blue), **1**·DMF (green) and **1**·DMA (orange).

Variable-temperature SCXRD revealed giant temperature-induced crystal deformation property for **1** (Fig. 3, Supplementary Fig. 3, Supplementary Tables 1 and 2 and Supplementary Data 1). While the *a*- and *c*-axes decrease by 2.2 and 1.6% from 112 to 300 K, respectively, its *b*-axis has a significant increase of 9.0%, giving rise to totally 5.8% increase of the unit-cell volume. Concomitantly, its void ratio increases from 20.4 to 24.8%. Such large temperature-induced crystal deformation has not been observed in coordination networks^15^,^16^,^17^,^18^,^19^,^25^. Comparison of the single-crystal structures at 112 and 300 K showed that, similar to other flexible PCPs, the framework distortions mainly arise from variation of the coordination angles rather than the coordination bonds (Supplementary Fig. 2 and Supplementary Tables 3 and 4). As defined by its **bcu** topology, the flexible 8-connected building unit can be regarded as a framework hinge and the whole framework can be considered as a 3D hinged rhombic fence consisting of many two-dimensional (2D) rhombic fences running across four crystallographic planes^15^,^53^. Although this framework topology is highly symmetric (cubic in its highest symmetric form), which allows the crystal to deform in any direction, the real deformation manner should depend on the anisotropy of the coordination network and pore system, as well as the distribution of the guest molecules. As the pores are running along the *a*-axis of **1**, it can be understood that the 3D coordination framework can distort easily on the *bc*-plane. Actually, there are 2D hinged fence-like motifs (consisting of dinuclear clusters and bent 34pba^–^ ligands) running across the *bc*-plane, whose distortion causes the acute expansion of the *b*-axis and simultaneous contraction of the *c*-axis, when the temperature increases. The relative small contraction of the *c*-axis can be attributed to the nonplanar shape of the *bc*-plane hinged fence, which transfers some contraction effect to the *a*-axis.

### Host–guest behaviour of the porous crystal

Gas adsorption measurements at low temperatures (Supplementary Fig. 4) showed that while **1** cannot adsorb N_2_ and Ar with kinetic diameters of 3.64 and 3.54 Å, respectively, at 77 K, it can readily adsorb the smaller O_2_ (3.47 Å) at the same condition. Nevertheless, the O_2_ isotherm exhibits obvious hysteresis and the saturated uptake (32 cm^3^ g^−1^) is significantly lower than the value empirically calculated from the crystal structure (146 cm^3^ g^−1^), indicating that the adsorption/desorption is very slow due to the high-energy barrier for gas diffusion in the quasi-0D ultramicropores. On the other hand, being similar with some flexible porous coordination polymers^3^,^16^, **1** can reversibly adsorb/release *N,N*-dimethylformamide (DMF) and *N,N*-dimethylacetamide (DMA) vapour at room temperature to give [Cd(34pba)(44pba)]·DMF (**1**·DMF) and [Cd(34pba)(44pba)]·DMA (**1**·DMA) without destroying the single-crystallinity (Supplementary Figs 5–7), indicating that the host framework possesses notable framework flexibility, especially for solvent molecules. SCXRD analyses of **1**·DMF and **1**·DMA at room temperature showed that they are isomorphic with **1**. Their unit-cell volumes are only 1.4% and 2.5% larger than that of **1** (Supplementary Fig. 1, Supplementary Table 1 and Supplementary Data 1), which are relatively small among flexible PCPs^2^,^3^,^4^. The most important structural feature of **1**·DMF and **1**·DMA is that each cavity is occupied by a pair of DMF/DMA guest molecules arranged in a centrosymmetric fashion with their molecular planes parallel with each other. Due to the size difference of the guests, the DMF and DMA supramolecular dimers are distinct in their configurations^54^,^55^. Specifically, the molecular plane of DMA has an inclined angle of 40.0° with the *bc*-plane, while that of DMF is only 17.3°, because the small pore forces the larger guest molecule to rotate to avoid significant steric hindrance. As a result of these molecular orientations, the DMF dimer exhibits much larger overlap as compared with those in the DMA dimer (Supplementary Fig. 8 and Supplementary Table 5). Consequently, adjacent DMA dimers form weak interaction (CH_3_···CH_3_ 4.15 Å), while the DMF dimers are well isolated from each other (shortest separation O···O 5.71 Å).

### Supramolecular-jack-like motion of the guest aggregations

Variable-temperature powder X-ray diffraction (PXRD) showed that (Fig. 3), while the (011) peaks of the three compounds gradually move to lower angles in different extents during temperature increase, the (100) peaks of **1** and **1**·DMF/**1**·DMA move in different directions, indicating that the thermal responses of their *a*-axes contrast with each other. SCXRD revealed that (Fig. 3, Supplementary Fig. 3, Supplementary Tables 1 and 2 and Supplementary Data 1), while the *a*-axis of **1** decreases by 2.2% from 112 to 300 K, those of **1**·DMF and **1**·DMA increase by 2.8 and 0.7%, respectively. Moreover, the unit-cell volume and the *b*-/*c*-axis of **1** changed more largely than those of **1**·DMF and **1**·DMA.

The restricted hinge motion of the *bc*-plane can be explained by the intrinsic steric hindrance effects of the guest molecules. When the quasi-0D cavities, that is, the apertures of the 2D hinged fences on the *bc*-plane, are filled by guest molecules, the flexibility of the hinged fence is reduced, giving smaller temperature-induced deformation on the *b*-/*c*-axis. Though DMF is smaller than DMA, the temperature-induced hinge motion on the *bc*-plane of **1**·DMF is the smallest one among the three materials, because the DMF molecules are almost parallel with the *bc*-plane, which exhibit strong steric hindrance effect with the host framework and restrain the hinge action across this plane (Supplementary Fig. 9).

**Figure 4:**
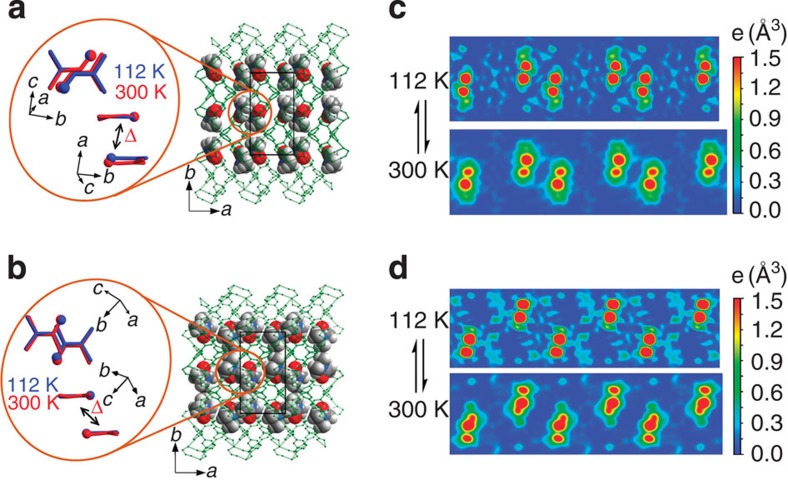
Temperature-induced structural transformations of the guest supramolecular aggregations. (**a**,**b**) Crystal structures of (**a**) **1**·DMF and (**b**) **1**·DMA. The host frameworks and the guest molecules are shown in green stick and multicolour space-filling modes (carbon, grey; nitrogen, blue; oxygen, red; hydrogen atoms are omitted for clarity), respectively. The enlarged insets compare the relative positions of the two guest molecules (stick mode with oxygen atoms highlighted as spheres) within the supramolecular dimer at 112 (blue) and 300 (red) K, along two representative directions (perpendicular and parallel to the molecular planes). The double-headed arrows represent the vectors of the guest–guest movements from 112 to 300 K within each dimer. (**c**,**d**) Temperature-dependent electron density maps of (**c**) **1**·DMF and (**d**) **1**·DMA.

More importantly, the reversed directions of changes of the *a*-axis can be assigned to the thermal expansion of the guest supramolecular aggregation (Fig. 4, Supplementary Figs 8 and 10 and Supplementary Table 6). In **1**·DMF, the interplanar separation within a dimer significantly increases by 0.45 Å from 112 to 300 K, while this value is only 0.15 Å in **1**·DMA, which highlights that the thermal expansion of the guest aggregation mainly occurs at the directions of the supramolecular interactions, and the tighter supramolecular contacts between the guest molecules can more effectively response to their local thermal vibrations. Comparison of their temperature-dependent electron density maps can clearly illustrate dynamic behaviours of the guest molecules. On the other hand, the closest interdimer separation decreases by 0.09 Å in **1**·DMF but increases by 0.17 Å in **1**·DMA. Since the interdimer interaction is ignorable in the crystal **1**·DMF, the separation does not expand with increasing temperature. Instead, the large thermal expansion within the DMF dimer reduces the interdimer separation. Nevertheless, the thermal expansion of the dimer mainly transfers to or expands the flexible host framework via host–guest interaction, so that the interdimer separation decreases by much <0.45 Å. In the case of **1**·DMA, the interdimer supramolecular contact is close enough to show thermal expansion, and the intradimer thermal expansion is not significant enough to compress the interdimer separation. As defined by the directions of the guest–guest interactions, the thermal expansions of the guests are highly anisotropic in the crystals, which is relatively parallel with the *a*-axis of **1**·DMF or has a considerable inclined angle with the *a*-axis of **1**·DMA. Therefore, the *a*-axis of **1**·DMA expands much less than **1**·DMF, since the thermal expansion of DMA supramolecular aggregations contribute to both the *a*- and *b*-axes. What's more, the void volume of **1**·DMF (from 23.4 to 27.0%) has a larger increase than that of **1**·DMA (from 27.5 to 28.8%), further indicating that the DMF dimer with much larger overlap can have stronger thermal expansion effect. While the static effects (that is, steric hindrance and supramolecular attraction to the host) of individual guest molecules have been observed to affect the thermal expansion behaviours of a few host frameworks^18^,^40^,^41^, this is the first structural evidence of thermal expansion of guest aggregations, which performs like supramolecular jacks to force the deformation of the host framework. It should be also mentioned that the relatively low crystallographic symmetry and suitable pore size (for a pair of amide molecules), as well as the multimode framework flexibility of **1** are decisive for the unambiguous observation of these interesting phenomena.

### Principal axial thermal expansion of the crystal

Since **1**, **1**·DMF and **1**·DMA crystallize in the monoclinic crystal system, the axial thermal expansion coefficients of the principal axes were calculated using the programme *PASCal* (Table 1 and Supplementary Table 7)^56^. The principal X_2_-axis is the same as the original crystallographic *b*-axis, while the principal X_1_- and X_3_-axes approximate the original crystallographic *a*- and *c*-axes or the [101] and [−101] directions. All the unit-cell parameters of **1**, **1**·DMF and **1**·DMA change linearly or approximately linearly against temperature, meaning that their axial thermal expansion coefficients are virtually constant in the measured temperature range. Such behaviour is critical for precise thermomechanical actuators and sensors. **1**·DMF shows huge PTE along the X_1_-axis (*α*_1_=+171 × 10^−6^ K^−1^) and moderate PTE and NTE along the X_2_- and X_3_-axes (*α*_2_=+60 × 10^−6^ K^−1^, *α*_3_=−56 × 10^−6^ K^−1^), respectively. When DMF is replaced by DMA, the thermal expansion coefficients of the X_1_- and X_2_-axes are approximately halved and doubled (*α*_1_=+85 × 10^−6^ K^−1^, *α*_2_=+103 × 10^−6^ K^−1^), respectively, while that of the X_3_-axis is almost unchanged (*α*_3_=−51 × 10^−6^ K^−1^). More remarkably, although the PTE of the X_1_-axis of **1** (*α*_1_=+61 × 10^−6^ K^−1^) is smaller than those of **1**·DMF and **1**·DMA, its X_2_- and X_3_-axes exhibit giant PTE of *α*_2_=+482 × 10^−6^ K^−1^ and NTE of *α*_3_=−218 × 10^−6^ K^−1^, respectively, which are much larger than for other known crystalline materials (Supplementary Table 8). It is noteworthy that the guest-modulation effect among **1**, **1**·DMF and **1**·DMA are exceptionally enormous, which change the PTE and NTE magnitudes by up to 422 × 10^−6^ K^−1^ and 167 × 10^−6^ K^−1^, respectively. Also interestingly, the X_2_-axial PTE coefficient follows the order **1**>**1**·DMA>**1**·DMF, while the X_1_-axial PTE coefficient follows the opposite order **1**·DMF>**1**·DMA>**1**. Consequently, the thermal expansion coefficients of the X_2_- and X_3_-axes of **1** are much larger than that of its X_1_-axis, whereas in **1**·DMF, the X_1_-axis shows the largest thermal expansion coefficient among three principal axes, meaning that the thermal expansion of the guest dimer can change the main thermal expansion axis or thermal expansion anisotropy of the crystal. Anisotropic physical property, as one of the most important features of crystalline materials, is predominantly determined by the crystallographic symmetry. The guest-dependent anisotropy of thermal expansion of isomorphic **1**, **1**·DMF and **1**·DMA (same crystallographic symmetry) demonstrates the possibility of controlling and utilizing the host–guest interplay for developing multi-responsive smart materials.

## Discussion

In summary, by using mixed linear and bent ligands, we successfully constructed an ultramicroporous flexible framework with a highly connected 3D hinge-like structure and multimode flexibility, which exhibits the largest PTE and NTE coefficients among framework solids. Remarkably, the quasi-0D ultramicropores in this material can accommodate dimers of different small amide molecules, giving unprecedentedly significant guest-modulation effects on both the magnitude and anisotropy of thermal expansion. By virtue of the robustness, low symmetry and suitable pore size of the crystal, the guest- and temperature-induced structural transformations were directly visualized by PXRD and SCXRD, which revealed that the confined guest dimers can show anisotropic thermal expansion behaviours similar with their bulk forms and force the host framework to drastically change not only the coefficients but also the main axis of thermal expansion. Generally, since the structural transformations of the host framework and the guest aggregations are always observed simultaneously, there is still no rational approach to analyse their relationship or identify which one is the original force. In this context, the multimode framework flexibility of the host framework, which has potential to deform in different ways under different stimuli, is crucial for unambiguous elucidation of the mechanism. Of course, these supramolecular-jack-like dimers and similar guest aggregations should be also effective and applicable for other flexible porous solids, even with only single-mode flexibility.

## Methods

### Materials

The ligands 3-(pyridin-4-yl)benzoic acid (H34pba) and 4-(pyridin-4-yl)benzoic acid (H44pba) were synthesized according to literature^17^,^32^. Other reagents were commercially available and used without further purification.

### Measurements

Elemental analyses were performed using a Vario EL elemental analyser. The FT-IR (KBr pellet) spectra were recorded in the range of 400–4,000 cm^−1^ on a Bruker TENSOR 27 FT-IR spectrometer. Thermogravimetric analyses were carried out using a TA Instruments Q50 thermogravimetric analyser under N_2_ at a rate of 10 °C/min. PXRD patterns were recorded on a Bruker D8-Advance diffractometer using Cu Kα radiation and a LynxEye detector at room temperature except otherwise stated. Gas sorption isotherms were measured on a volumetric adsorption apparatus (Micromeritics ASAP 2020 M Physisorption Analyzer). Ultrahigh-purity-grade (purity>99.999%) N_2_, O_2_ and Ar gases were used in all adsorption measurements. As-synthesized samples were degassed under high vacuum at 493 K for 3 h before measurement. Temperatures were maintained by liquid nitrogen.

### Synthesis

H34pba (0.40 g, 2.0 mmol) and H44pba (0.40 g, 2.0 mmol) were dissolved in EtOH (80 ml) using a 250-ml scintillation vial, added with a water solution of Cd(NO_3_)_2_ (0.05 mol l^−1^, 40 ml). The mixture was then sealed with a screw cap and heated to 90 °C for 24 h. Colourless needle-like crystals of **1**·EtOH·H_2_O were obtained (yielded 0.81 g, ∼80%). Then the samples were filtered and briefly dried in air to give crystals of **1**. Elemental analysis calcd (%) for [Cd(34pba)(44pba)]·3H_2_O (C_24_H_21_N_2_O_7_Cd): C 51.31, H 3.77, N 4.99; found: C 51.20, H 4.35, N 4.68. FT-IR (KBr, cm^−1^): 3,426(m), 3,062(w), 2,920(w), 1,610(s), 1,556(s), 1,396(s), 1,276(w), 1,224(w), 1,072(w), 1,014(w), 858(m), 837(m), 765(m), 736(m), 678(m), 624(w), 559(w) and 503(m). When single crystals of **1** were sealed in a test tube saturated with DMF/DMA vapour for 3 days, some single crystals of **1**·DMF/**1**·DMA were obtained.

### X-ray single-crystal structure analyses

Diffraction data were collected on an Agilent SuperNova CCD diffractometer with graphite-monochromated Cu Kα radiation. Single crystals of **1**·DMF and **1**·DMA were mounted directly on the top of a glass fibre using minimum amount of glue. A single crystal of **1** was sealed in high vacuum in a glass capillary. The test temperature was controlled by dry nitrogen flow using a Cryostream Plus cooler system, and corrected by a thermal couple at the crystal position. Though thermogravimetry curves of **1**·DMF and **1**·DMA showed no weight loss below 350 K, the unit-cell parameters of **1**·DMF and **1**·DMA did change above 340 K under the temperature-controlling nitrogen flow after ∼2 h, which can be attributed to the guest escaping. Nevertheless, under the same measurement conditions, no change of unit-cell parameter or chemical composition occurred for single crystals of **1**·DMF and **1**·DMA below 300 K even for one day. The unit-cell parameters at each measurement temperature used for calculation of the thermal expansion coefficients were determined by 30 diffraction images, while those for crystal-structure refinements were determined by 600–900 diffraction images. The reversibility of temperature-induced crystal deformations of the samples were confirmed by the coincidence of data points over a heating–cooling cycle. Absorption corrections were applied by using the multi-scan programme *CrysAlisPro*^57^. The crystal structures were solved through direct methods and developed by difference Fourier techniques using the *SHELXTL* software packages^58^. All hydrogen atoms were added geometrically, and anisotropic displacement parameters were used to refine all non-hydrogen atoms.

## Author contributions

J.P.Z. designed the research; H.L.Z. performed synthesis and measurements; J.P.Z. and H.L.Z. analysed data; all authors discussed the results, contributed to the writing of manuscript and commented on it.

## Additional information

**Accession codes.** The X-ray crystallographic coordinates for structures reported in this Article have been deposited at the Cambridge Crystallographic Data Centre (CCDC), under deposition number CCDC 1049147–1049152. These data can be obtained free of charge from The Cambridge Crystallographic Data Centre via www.ccdc.cam.ac.uk/data_request/cif.

**How to cite this article:** Zhou, H.-L. *et al*. Supramolecular-jack-like guest in ultramicroporous crystal for exceptional thermal expansion behaviour. *Nat. Commun*. 6:6917 doi: 10.1038/ncomms7917 (2015).

## Supplementary Material

Supplementary Figures, Supplementary Tables and Supplementary References Supplementary Figures 1-10, Supplementary Tables 1-8 and Supplementary References.

Supplementary Data 1 X-ray single-crystal structures in CIF format

## Figures and Tables

**Figure 1:**
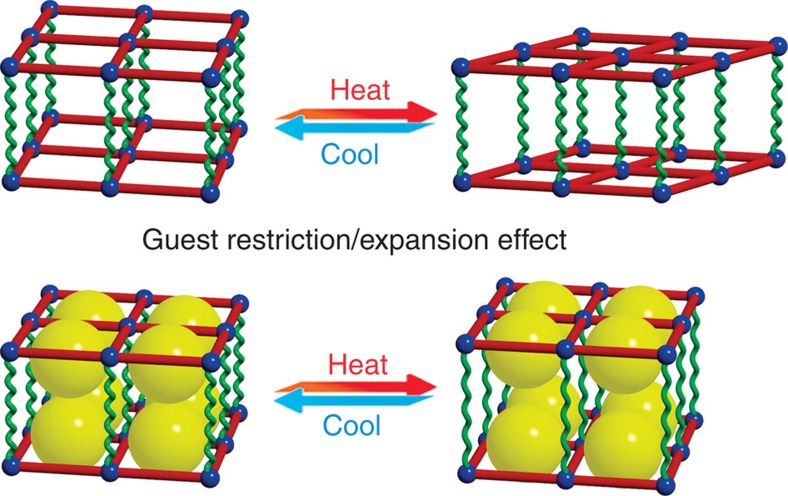
Guest-controlled multimode framework flexibility. The red squares and green spires represent two possible responsive modes in a porous crystal encoded with multimode framework flexibility. Without guest, thermal expansion of the host framework is mainly determined by the hinge action across the red fences, which can be restricted by the steric hindrance effect of guest (yellow spheres). Further, the anisotropic thermal expansion effect of the guest dimers (significant change of separation between two yellow spheres), behaving like a jack, can force the host framework to deform in another direction along the green spires.

**Table 1:** Temperature-/guest-dependent thermal expansion coefficients.

Compound	Principal axis	Direction	*α* ( × 10^−6^ K^−1^)	*β*_*V*_ ( × 10^−6^ K^−1^)
**1**	X_1_	∼*a*	+61(1)	+319(13)
	X_2_	*b*	+482(12)	
	X_3_	∼*c*	−218(3)	
**1**·DMF	X_1_	∼*a*	+171(4)	+175(2)
	X_2_	*b*	+60(3)	
	X_3_	∼*c*	−56(2)	
**1**·DMA	X_1_	∼*a*	+85(1)	+138(2)
	X_2_	*b*	+103(4)	
	X_3_	∼*c*	−51(3)	

α and β_*V*_ represent axial and volumetric thermal expansion coefficients, respectively.
