# Supplementary material for: Supramolecular-jack-like guest in ultramicroporous crystal for exceptional thermal expansion behaviour
Source: Nat Commun. 2015 Apr 21;6:6917. doi: 10.1038/ncomms7917 (PMC4411299; doi:10.1038/ncomms7917)
Supplement: Supplementary Figures, Supplementary Tables and Supplementary References — Supplementary Figures 1-10, Supplementary Tables 1-8 and Supplementary References. [file ncomms7917-s1.pdf]

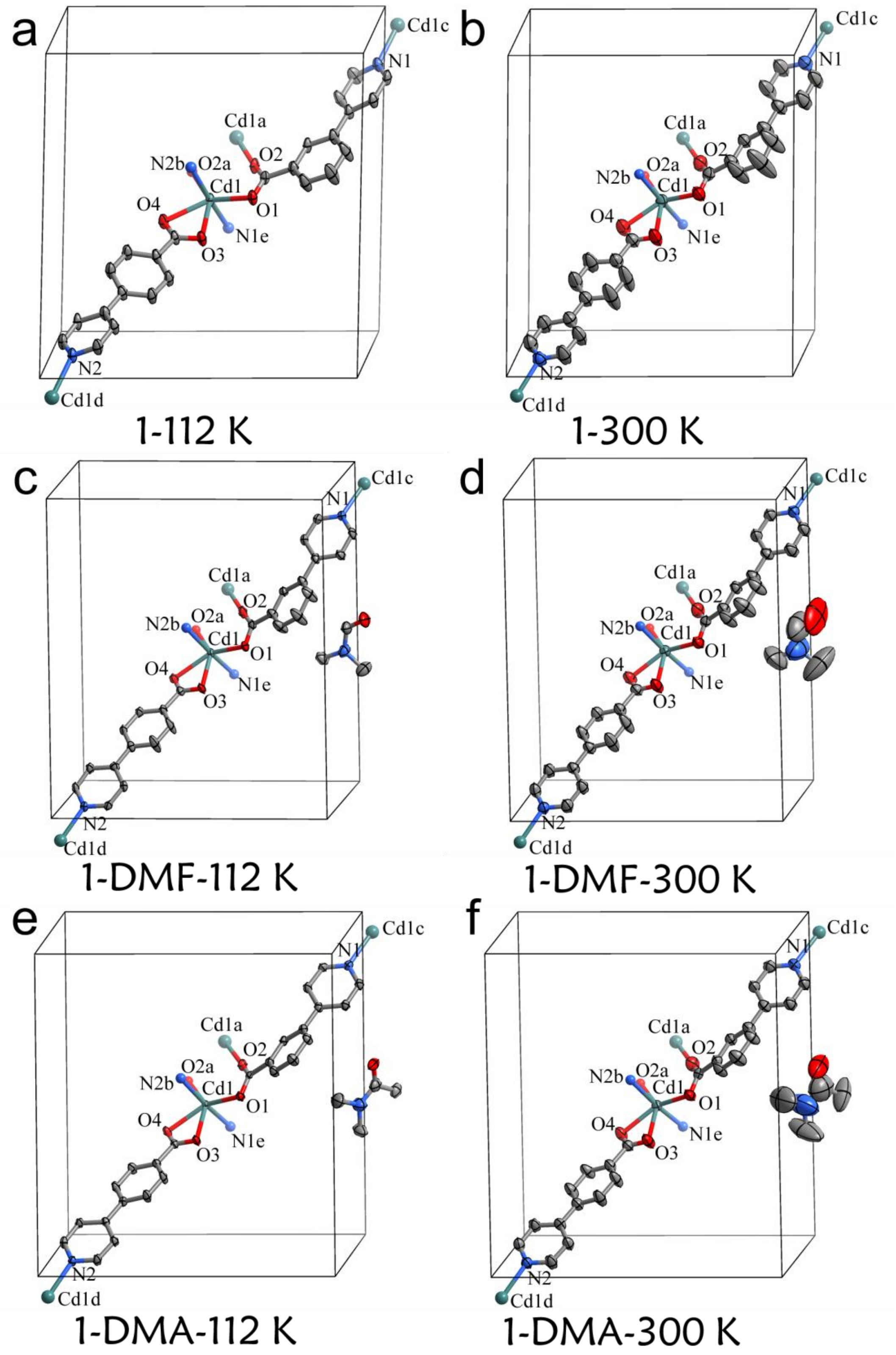

Supplementary Figure 1 Ortep plot of (a, b) **1** at 112 K and 300 K, (c, d) **1**·DMF at 112 K and 300 K, and (e, f) **1**·DMA at 112 K and 300 K. Thermal ellipsoids are drawn at 50% probability. Hydrogen atoms are omitted for clarity. Symmetry Codes: a = 2-x, 1-y, 1-z; b = 1-x, -1/2+y, 3/2-z; c = 2-x, -1/2+y, 1/2-z; d = 1-x, 1/2+y, 3/2-z; e = 2-x, 1/2+y, 1/2-z.

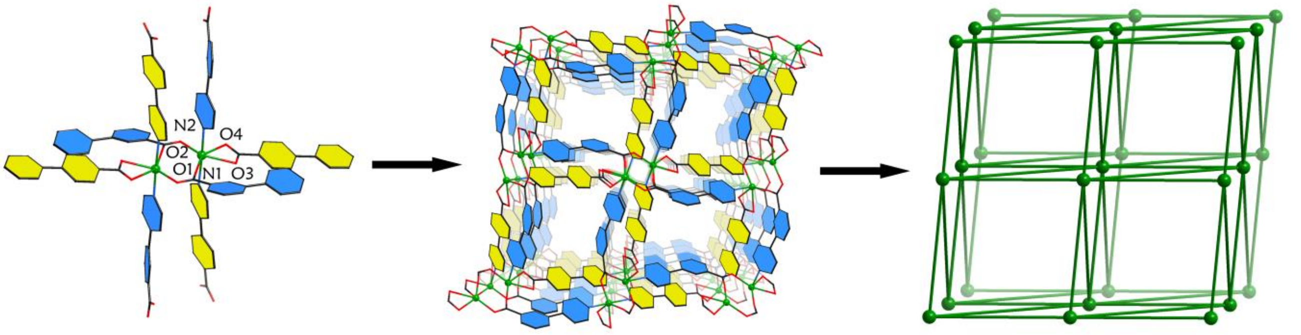

Supplementary Figure 2 Simplification of the coordination framework of **1** into an 8-connected **bcu** net. Codenames are given for selected atoms in the dinuclear cluster, which will be quoted for structural detail comparison of **1**, **1**·DMF and **1**·DMA.

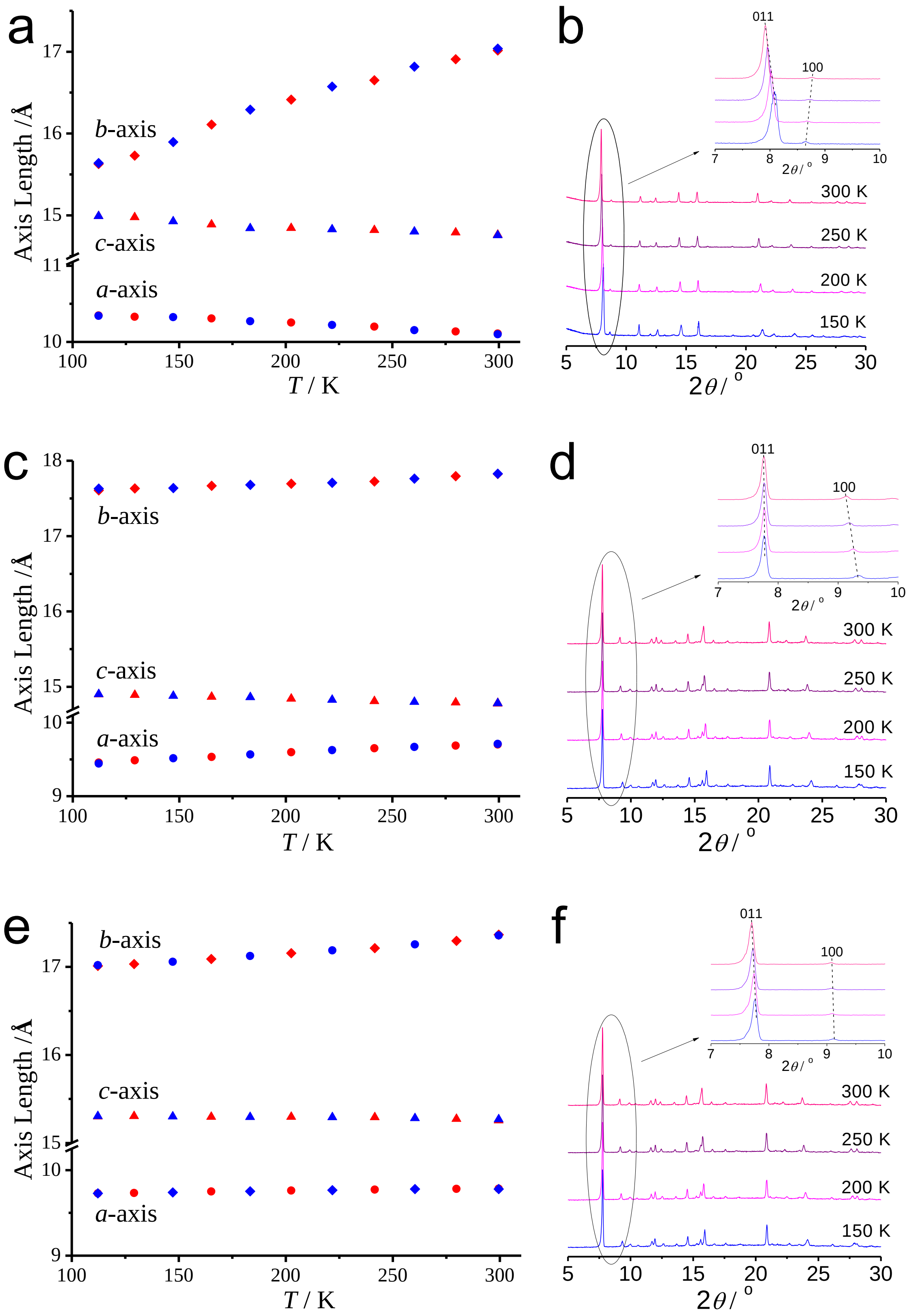

Supplementary Figure 3 The heating (red) and cooling (blue) history of temperature dependence of the unit-cell parameters and variable-temperature PXRD patterns of (a,b) **1**, (c,d) **1**·DMF, and (e,f) **1**·DMA.

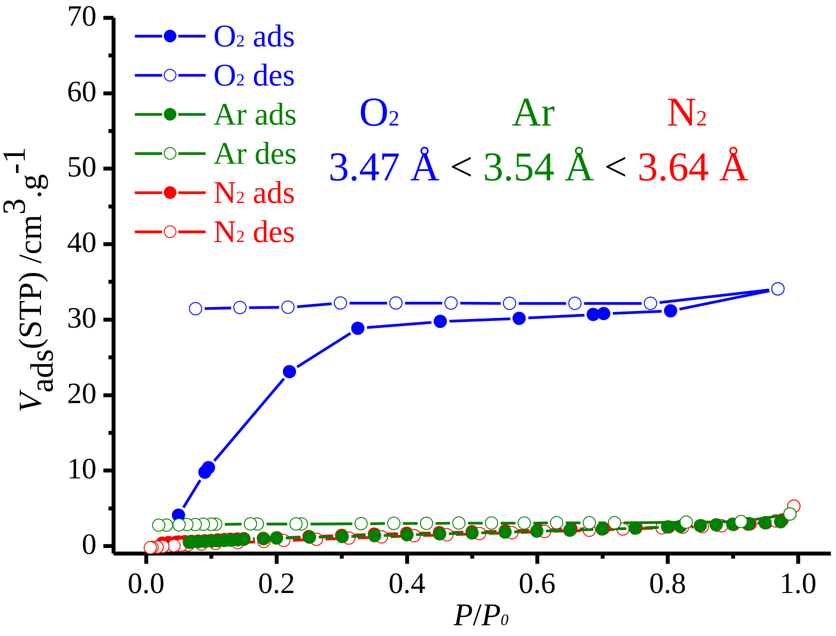

Supplementary Figure 4 Gas adsorption isotherms of **1** at 77 K.

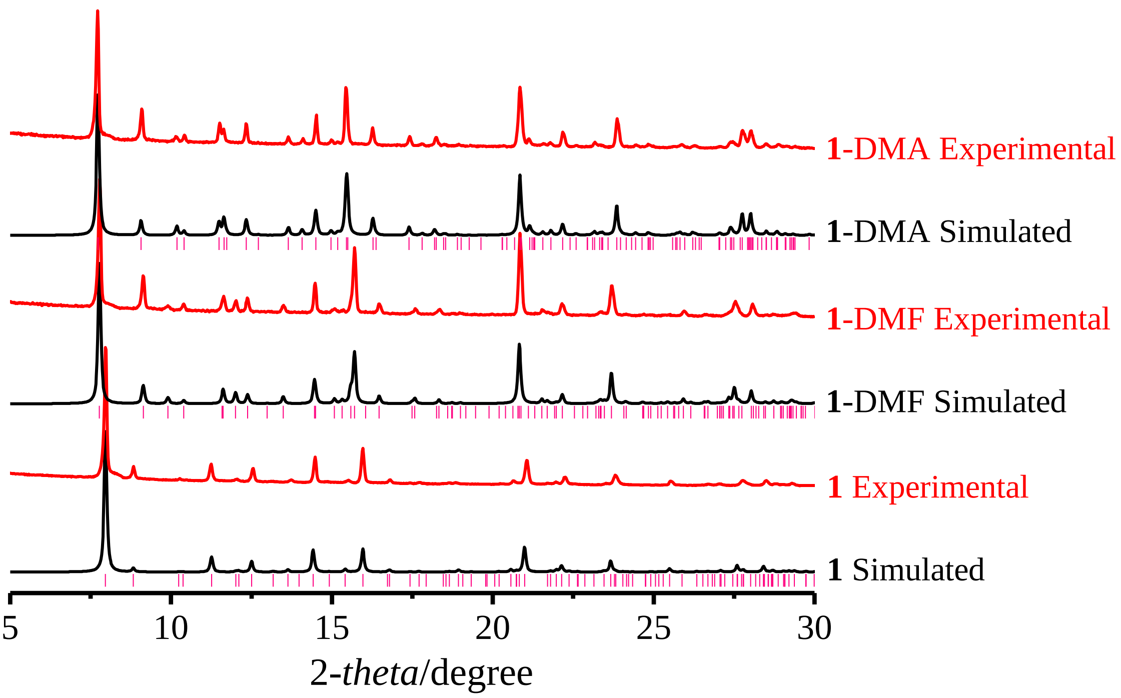

Supplementary Figure 5 PXRD patterns of **1**, **1**·DMF and **1**·DMA.

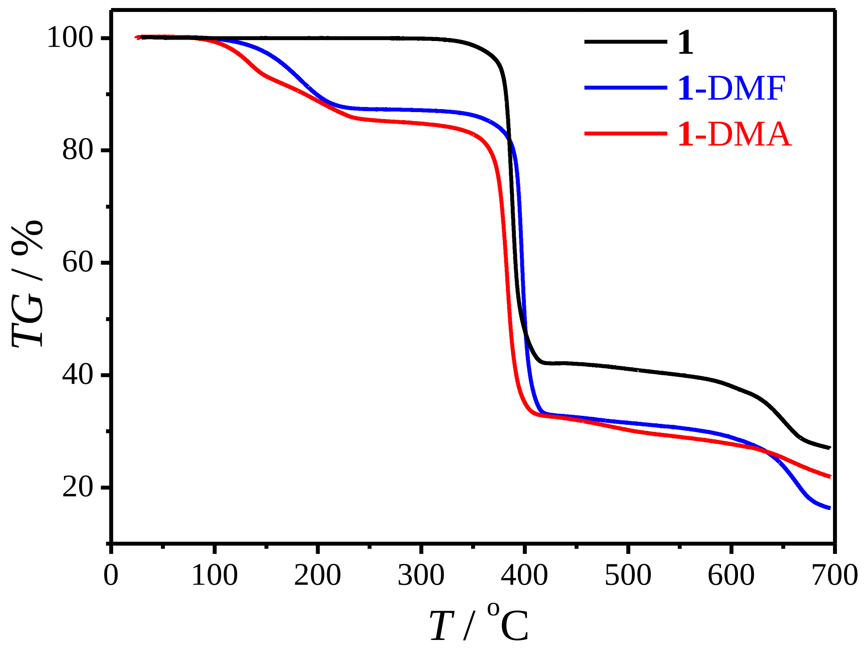

Supplementary Figure 6 TG curves of **1**, **1**·DMF and **1**·DMA. The guests start to escape from **1**·DMF and **1**·DMA at 100 °C and 80 °C, respectively. Complete release of the included guest molecules for **1**·DMF (calcd: 12.6%, observed 12.7%) and **1**·DMA (calcd: 14.6%, observed 14.8%) occur at 230 °C and 250 °C, respectively.

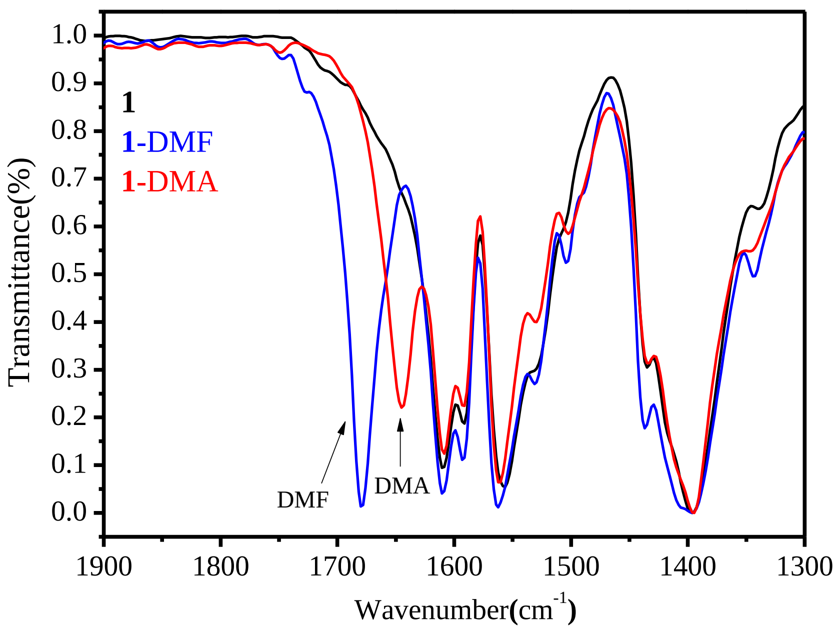

Supplementary Figure 7 IR spectra of **1**, **1**·DMF and **1**·DMA.

a

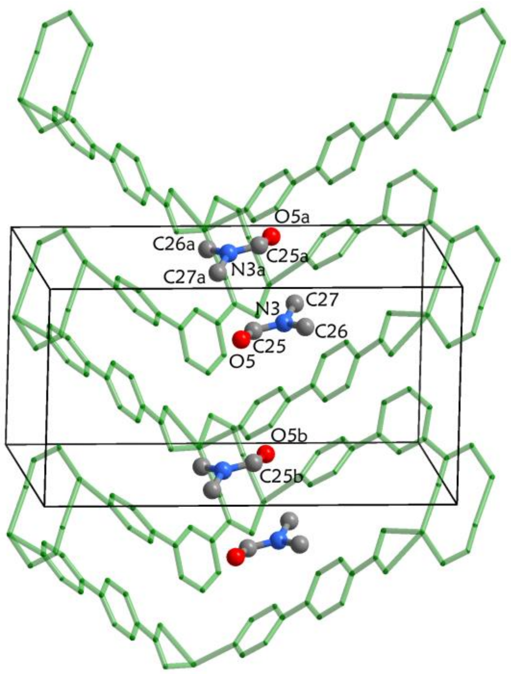

b

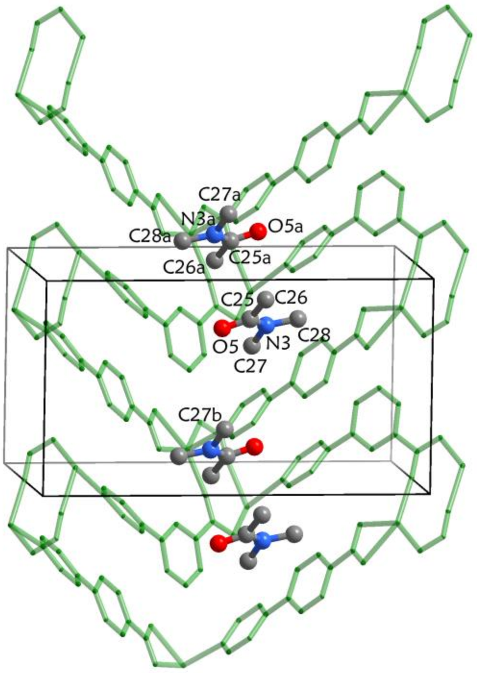

Supplementary Figure 8 Codenames of independent atoms of the guest dimers for structural detail comparison of (a) **1**·DMF and (b) **1**·DMA. Symmetry Codes: a = 2-x, 1-y, -z; b = 1-x, 1-y, -z.

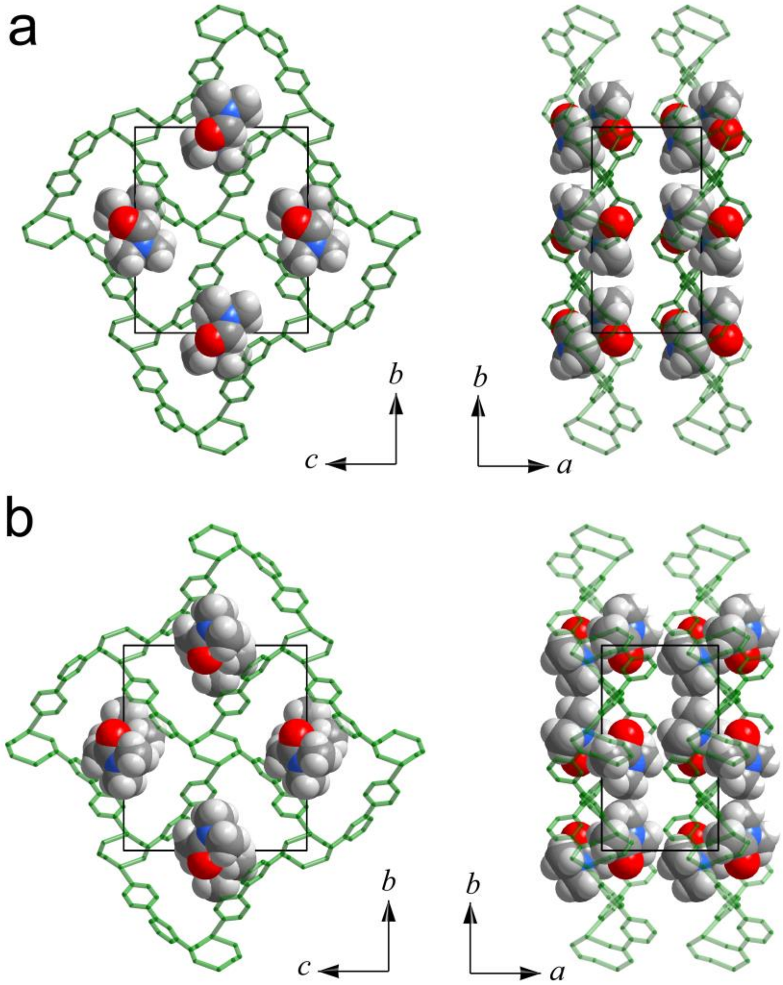

Supplementary Figure 9 Perspective view of the hinged fence on the *ab*-plane and the position of the guest dimers of (a) **1**·DMF and (b) **1**·DMA along the *a*-axis. The host frameworks and the guest molecules are shown in green stick and multicolor space-filling modes (carbon, grey; nitrogen, blue; oxygen, red), respectively.

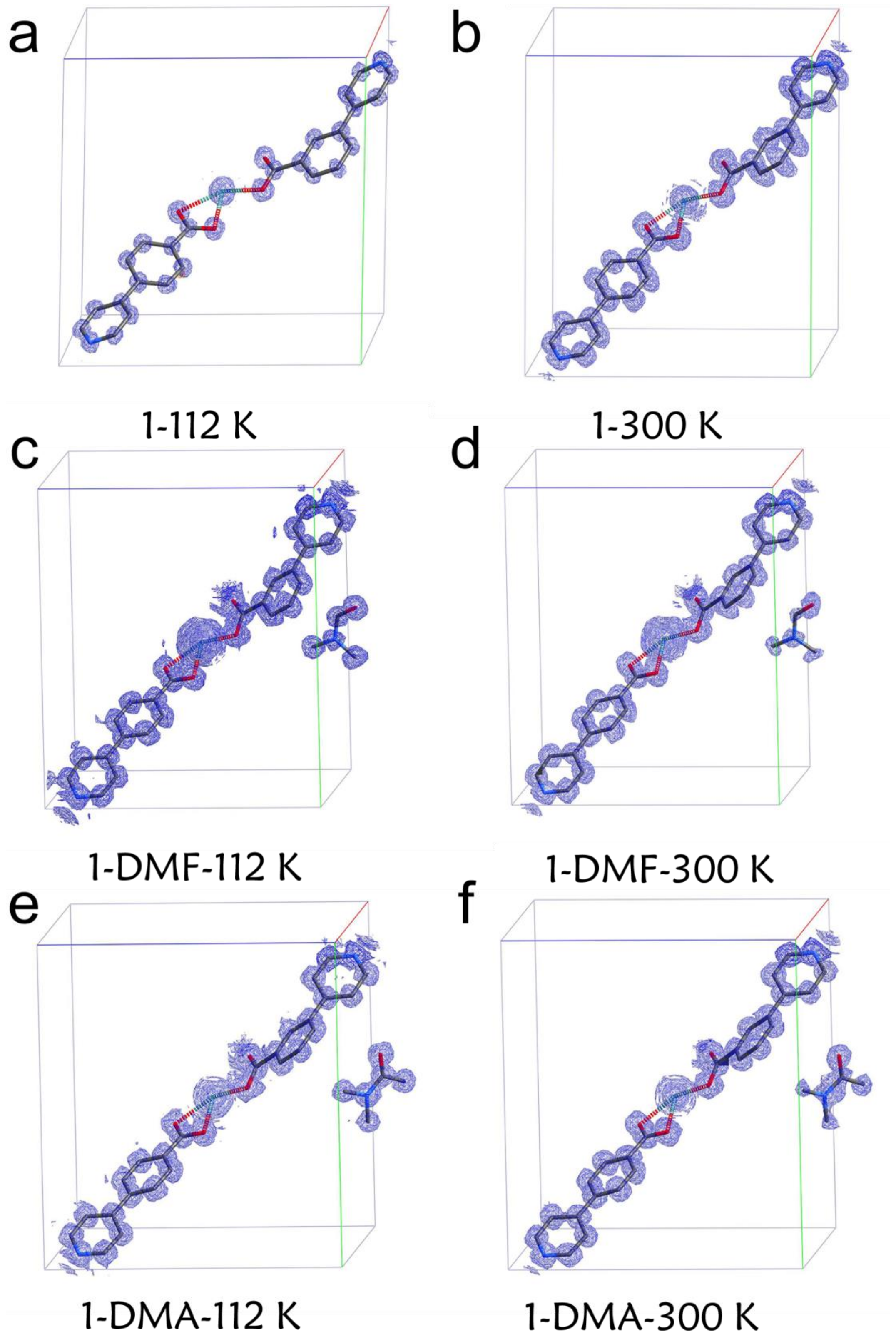

Supplementary Figure 10 Electron density (F_{obs}) map of (a, b) **1** at 112 K and 300 K, (c, d) **1**·DMF at 112 K and 300 K, and (e, f) **1**·DMA at 112 K and 300 K. The settings of the *ShelXle* program: map precision = 3; factor to down weight weak data = 1; *F*-observed map = 1.00 e Å^{-3}; map truncation type = 1.41 Å around visible atoms or peaks; line transparency 0.6; line width = 1.0.

Supplementary Table 1 Crystallographic Data and Structural Refinements.

Complex	**1**-112K	**1**-300K	**1**·DMF-112K	**1**·DMF-300K	**1**·DMA-112K	**1**·DMA-300K
Formula	$C_{24}H_{16}CdN_2O_4$	$C_{24}H_{16}CdN_2O_4$	$C_{27}H_{23}CdN_3O_5$	$C_{27}H_{23}CdN_3O_5$	$C_{28}H_{25}CdN_3O_5$	$C_{28}H_{25}CdN_3O_5$
Formula	508.79	508.79	581.88	581.88	595.91	595.91
Temperature/K	112(2)	300(2)	112(2)	300(2)	112(2)	300(2)
Crystal system	Monoclinic	Monoclinic	Monoclinic	Monoclinic	Monoclinic	Monoclinic
Space group	$P2_1/c$	$P2_1/c$	$P2_1/c$	$P2_1/c$	$P2_1/c$	$P2_1/c$
a/Å	10.3397(3)	10.10785(19)	9.4420(3)	9.70550(15)	9.72700(18)	9.79040(15)
b/Å	15.6399(5)	17.0515(4)	17.6271(5)	17.8498(3)	17.0204(3)	17.3660(3)
c/Å	14.9889(4)	14.7514(3)	14.8977(4)	14.7845(3)	15.2959(2)	15.2569(2)
$\beta/^{o}$	100.948(3)	97.9248(17)	96.085(3)	94.7485(15)	96.8477(17)	95.4829(13)
V/Å^3	2379.76(11)	2518.17(9)	2465.53(13)	2552.50(8)	2514.28(8)	2582.11(7)
Z	4	4	4	4	4	4
D_c /g cm^{-3}	1.420	1.342	1.568	1.514	1.574	1.533
reflns coll.	7898	8438	8316	8443	8148	8319
unique reflns	4343	4698	4606	4788	4712	4818
R_{int}	0.0279	0.0289	0.0220	0.0237	0.0204	0.0190
R_1 $[I > 2\sigma(I)]$[a]	0.0483	0.0456	0.0434	0.0374	0.0315	0.0320
wR_2 $[I > 2\sigma(I)]$[b]	0.1321	0.1282	0.1178	0.0978	0.0798	0.0848
R_1 (all data)	0.0544	0.0552	0.0465	0.0410	0.0351	0.0350
wR_2 (all data)	0.1427	0.1407	0.1203	0.1012	0.0827	0.0877
GOF	1.039	1.066	1.052	1.043	1.060	1.046
Void/%	20.4	24.8	23.4	27.0	27.5	28.8

$^{a}R_1=\Sigma||F_o| - |F_c||/\Sigma|F_o|$. $^{b}wR_2 = [\Sigma w(F_o^2 - F_c^2)^2/\Sigma w(F_o^2)^2]^{1/2}$.

Supplementary Table 2 Temperature-dependent unit-cell parameters of **1**, **1**·DMF, and **1**·DMF.

**1**					
T (K)	*a* (Å)	*b* (Å)	*c* (Å)	*β* (°)	*V* (Å^3)
299.45	10.099(4)	17.037(5)	14.757(4)	98.05(3)	2514(1)
260.35	10.154(4)	16.818(5)	14.802(3)	98.64(3)	2499(1)
221.75	10.223(5)	16.573(5)	14.828(3)	99.26(4)	2480(1)
183.35	10.270(4)	16.291(5)	14.842(4)	99.80(4)	2447(1)
147.15	10.324(5)	15.896(7)	14.927(5)	100.58(5)	2408(2)
112.15	10.341(5)	15.638(6)	14.991(5)	101.04(5)	2379(2)
112.15	10.347(4)	15.627(5)	14.990(4)	101.07(4)	2379(1)
129.15	10.330(5)	15.729(7)	14.976(5)	100.79(5)	2390(2)
165.15	10.307(5)	16.109(5)	14.888(4)	100.26(4)	2432(2)
202.55	10.253(4)	16.414(4)	14.844(3)	99.63(3)	2463(1)
241.55	10.201(5)	16.651(5)	14.820(4)	99.06(4)	2486(2)
279.75	10.137(4)	16.909(5)	14.789(4)	98.40(4)	2508(1)
299.45	10.110(4)	17.015(5)	14.762(4)	98.09(3)	2514(1)

Supplementary Table 2 Temperature-dependent unit-cell parameters of **1**, **1**·DMF, and **1**·DMF (continue).

**1**·DMF					
T (K)	*a* (Å)	*b* (Å)	*c* (Å)	β (º)	*V* (Å^3)
299.45	9.709(3)	17.827(3)	14.782(2)	94.81(2)	2549(1)
260.35	9.669(5)	17.763(4)	14.800(3)	95.09(3)	2532(2)
221.75	9.626(6)	17.707(5)	14.828(4)	95.45(3)	2516(2)
183.35	9.567(7)	17.680(5)	14.862(5)	95.74(4)	2501(2)
147.15	9.515(6)	17.637(5)	14.881(4)	95.89(3)	2484(2)
112.15	9.444(7)	17.629(6)	14.902(5)	96.23(4)	2466(2)
112.15	9.456(7)	17.607(5)	14.899(5)	96.14(4)	2466(2)
129.15	9.486(6)	17.631(5)	14.893(4)	96.05(3)	2477(2)
165.15	9.533(7)	17.667(5)	14.868(4)	95.81(4)	2491(2)
202.55	9.596(7)	17.695(6)	14.843(5)	95.57(4)	2509(2)
241.55	9.651(6)	17.725(5)	14.810(4)	95.32(3)	2522(2)
279.75	9.687(4)	17.795(3)	14.793(2)	94.98(2)	2541(1)
299.45	9.703(4)	17.825(4)	14.785(3)	94.84(2)	2548(1)

Supplementary Table 2 Temperature-dependent unit-cell parameters of **1**, **1**·DMF, and **1**·DMF (continue).

**1**·DMA					
T (K)	*a* (Å)	*b* (Å)	*c* (Å)	β (º)	*V* (Å^3)
299.45	9.778(3)	17.358(3)	15.268(4)	95.47(3)	2580(1)
260.35	9.7778(19)	17.2565(18)	15.282(3)	95.853(19)	2565.2(7)
221.75	9.765(2)	17.188(2)	15.292(4)	96.14(2)	2551.9(10)
183.35	9.753(2)	17.124(2)	15.295(4)	96.38(2)	2538.5(9)
147.15	9.740(2)	17.059(2)	15.300(4)	96.59(2)	2525.4(9)
112.15	9.727(2)	17.019(2)	15.305(4)	96.87(3)	2515.5(10)
112.15	9.728(2)	17.011(2)	15.300(4)	96.87(2)	2513.8(9)
129.15	9.732(2)	17.033(2)	15.306(4)	96.76(3)	2519.6(9)
165.15	9.749(2)	17.090(2)	15.297(4)	96.50(3)	2532.5(10)
202.55	9.7599(18)	17.1564(17)	15.296(3)	96.271(18)	2545.9(7)
241.55	9.771(2)	17.2133(19)	15.293(3)	95.97(2)	2558.0(8)
279.75	9.780(2)	17.296(2)	15.273(4)	95.71(2)	2570.8(10)
299.45	9.782(3)	17.367(3)	15.257(4)	95.50(3)	2580(1)

Supplementary Table 3 Coordination bond lengths (Å) of **1**, **1**·DMF and **1**·DMA at different temperatures (see Supplementary Fig. S2).

	**1**-112K	**1**-300K	**1**·DMF-112K	**1**·DMF-300K	**1**·DMA-112K	**1**·DMA-300K
Cd1-O1	2.319(3)	2.279(3)	2.288(3)	2.280(3)	2.295(2)	2.275(2)
Cd1-O3	2.335(3)	2.349(5)	2.351(3)	2.362(3)	2.349(2)	2.373(3)
Cd1-O4	2.434(3)	2.414(5)	2.436(3)	2.405(3)	2.427(2)	2.390(2)
Cd1-N2	2.306(4)	2.323(5)	2.299(3)	2.317(3)	2.306(3)	2.327(3)
Cd1-N1	2.289(4)	2.298(5)	2.295(4)	2.310(3)	2.301(3)	2.316(3)
Cd1-O2	2.310(3)	2.294(4)	2.305(3)	2.298(3)	2.321(2)	2.308(3)

Supplementary Table 4 Coordination bond angles (°) of **1**, **1**·DMF and **1**·DMA at different temperatures (see Supplementary Fig. S2).

	**1**-112K	**1**-300K	**1**·DMF-112K	**1**·DMF-300K	**1**·DMA-112K	**1**·DMA-300K
O1-Cd1-O3	82.87(11)	86.27(14)	88.77(10)	88.21(10)	87.14(7)	88.31(9)
O1-Cd1-O4	135.58(11)	140.31(14)	143.65(10)	142.77(10)	141.77(7)	142.93(7)
O1-Cd1-N2	88.99(11)	89.26(14)	92.52(11)	90.19(10)	88.10(8)	88.09(9)
O1-Cd1-N1	93.75(13)	94.00(14)	91.60(11)	92.58(10)	94.26(8)	93.72(9)
O1-Cd1-O2	131.05(11)	128.46(14)	129.92(11)	128.78(10)	131.84(8)	129.63(9)
O3-Cd1-O4	55.44(10)	54.75(15)	55.12(10)	54.74(10)	55.18(7)	54.94(8)
O3-Cd1-N2	93.79(13)	91.95(16)	92.93(12)	91.17(11)	90.21(9)	90.30(10)
O3-Cd1-N1	93.26(14)	93.56(16)	94.02(12)	95.32(10)	96.97(9)	97.17(10)
O2-Cd1-O3	146.07(10)	145.12(15)	141.30(11)	142.80(10)	140.52(7)	141.64(9)
O4-Cd1-N2	80.16(12)	85.37(16)	86.01(11)	87.35(10)	86.06(8)	87.51(9)
O4-Cd1-N1	102.39(13)	95.45(16)	94.72(11)	94.28(10)	96.37(8)	95.44(9)
O2-Cd1-O4	91.48(10)	90.40(15)	86.23(10)	88.06(10)	85.35(7)	86.73(8)
N1-Cd1-N2	172.71(15)	173.76(15)	172.00(13)	173.03(10)	172.53(10)	172.35(10)
O2-Cd1-N2	86.77(13)	86.00(14)	85.47(12)	85.61(11)	85.81(9)	86.16(10)
O2-Cd1-N1	86.34(13)	87.81(14)	86.63(12)	87.66(11)	87.34(9)	86.97(10)

Supplementary Table 5 Selected interdimer supramolecular contacts (Å) of **1**·DMF and **1**·DMA at different temperatures (see Supplementary Fig. S8).

**1**·DMF				**1**·DMA			
	112 K	300 K	*d*		112 K	300 K	*d*
C25-C25b	6.142(9)	5.90(2)	-0.24(2)	C27-C27b	3.978(8)	4.15(2)	0.17(2)
O5-O5b	5.797(7)	5.71(2)	-0.09(2)				
C25-O5b	5.845(8)	5.68(2)	-0.17(2)				

Supplementary Table 6 Selected intradimer supramolecular contacts (Å) of **1**·DMF and **1**·DMA at different temperatures (see Supplementary Fig. S8).

**1**·DMF				**1**·DMA			
	112 K	300 K	*d*		112 K	300 K	*d*
C25-C25a	3.37(1)	3.88(2)	0.51(2)	C25-C25a	3.531(7)	3.69(1)	0.16(2)
C25-N3a	3.486(8)	3.84(1)	0.35(2)	C25-N3a	4.054(7)	4.10(2)	0.05(2)
C25-O5a	3.723(8)	4.16(2)	0.46(2)	C25-O5a	3.856(6)	4.01(1)	0.15(2)
C25-C27a	3.851(9)	4.19(2)	0.34(2)	C25-C26a	3.391(6)	3.56(1)	0.17(2)
N3-C25a	3.486(8)	3.84(1)	0.35(2)	N3-C25a	4.054(7)	4.10(2)	0.05(2)
N3-N3a	4.067(7)	4.25(1)	0.18(2)	N3-O5a	4.144(6)	4.20(1)	0.06(2)
N3-O5a	3.571(7)	3.85(2)	0.28(2)	N3-C26a	3.707(6)	3.79(1)	0.08(2)
O5-C25a	3.723(8)	4.16(2)	0.46(2)	O5-C25a	3.856(6)	4.01(1)	0.15(2)
O5-N3a	3.571(7)	3.85(2)	0.28(2)	O5-N3a	4.144(6)	4.20(1)	0.06(2)
O5-O5a	4.403(7)	4.74(2)	0.34(2)	O5-C26a	3.440(6)	3.57(1)	0.13(2)
O5-C27a	3.445(8)	3.78(2)	0.34(2)	C26-C25a	3.391(6)	3.56(1)	0.17(2)
C27-C25a	3.851(9)	4.19(2)	0.34(2)	C26-N3a	3.707(6)	3.79(1)	0.08(2)
C27-O5a	3.445(8)	3.78(2)	0.34(2)	C26-O5a	3.440(6)	3.57(1)	0.13(2)
C27-C27a	5.50(1)	5.69(2)	0.19(2)	C26-C26a	3.882(7)	4.04(1)	0.16(2)

Supplementary Table 7 Relationship between crystallography axes and principal axes.

Compound	Principal axis	direction	Component of principal axis along the		
			a	*b*	*c*
**1**	X_1	~*a*	0.8371	0	0.5471
	X_2	*b*	0	1	0
	X_3	~*c*	-0.8014	0	0.5981
**1**·DMF	X_1	~*a*	0.9805	0	0.1963
	X_2	*b*	0	1	0
	X_3	~*c*	-0.3112	0	0.9470
**1**·DMA	X_1	~*a*	0.9099	0	0.4147
	X_2	*b*	0	1	0
	X_3	~*c*	-0.7170	0	0.6971

Supplementary Table 8 Axial thermal expansion coefficients of representative solid materials.

Compound	$\alpha_{PTE}/\times10^{-6}$ K^{-1}	$\alpha_{NTE}/\times10^{-6}$ K^{-1}	Reference
ZrW_2O_8	NA	−9.1	1
$Ag_3[Co(CN)_6]$	150	−130	2
(*S*,*S*)-Octa-3,5-diyn-2,7-diol	+156 ~ +515 [a]	−48 ~ −204 [a]	3
MCF-18·DMF	+81 ~ +437 [a]	NA	4
MCF-18·MeOH	+12 ~ +242[a]	NA	4
MCF-18	+81	NA	4
$[Ag(en)]NO_3$-I	+149	−90	5
PHA-α	+260	−80	6
Cd(im)	+93	−23	7
Ag(mim)	+130	−25	8
FMOF-1	+230	−170	9
HMOF-1	+177	NA	10
[Zn(OH)(niba)]	+137	NA	11
[Zn(OH)(niba)]·MeOH	+166	NA	11
[Zn(OH)(niba)]·*i*-PrOH	+76	NA	11
MCF-34	+224	−107	12
MCF-34·DMF	+152 or +237 [b]	−56 or −116 [b]	12
**1** (MCF-82)	+482	−218	This work

[a] The unit-cell parameters change nonlinearly against temperature, *i.e.* inconstant thermal expansion coefficients.

[b] Thermal expansion coefficients of low-temperature and high-temperature phases, respectively. The crystal deformation during phase transition is not regarded as thermal expansion.

NA: Not applicable, no data, or very small.

References

1. Mary, T. A., Evans, J. S. O., Vogt, T. & Sleight, A. W. Negative Thermal Expansion from 0.3 to 1050 Kelvin in ZrW_2O_8. *Science* **272**, 90-92 (1996).

2. Goodwin, A. L., Calleja, M., Conterio, M. J., Dove, M. T., Evans, J. S. O., Keen, D. A., Peters, L. & Tucker, M. G. Colossal Positive and Negative Thermal Expansion in the Framework Material $Ag_3[Co(CN)_6]$. *Science* **319**, 794-797 (2008).

3. Das, D., Jacobs, T. & Barbour, L. J. Exceptionally large positive and negative anisotropic thermal expansion of an organic crystalline material. *Nat. Mater.* **9**, 36-39 (2010).

4. Wei, Y.-S., Chen, K.-J., Liao, P.-Q., Zhu, B.-Y., Lin, R.-B., Zhou, H.-L., Wang, B.-Y., Xue, W., Zhang, J.-P. & Chen, X.-M. Turning on the flexibility of isoreticular porous coordination frameworks for drastically tunable framework breathing and thermal expansion. *Chem. Sci.* **9**, 1539-1546 (2013).

5. Cai, W. & Katrusiak, A. Giant negative linear compression positively coupled to massive thermal expansion in a metal-organic framework. *Nat. Commun.* **5**, 4337 (2014).

6. Panda, M. K., Runčevski, T., Chandra Sahoo, S., Belik, A. A., Nath, N. K., Dinnebier, R. E. & Naumov, P. Colossal positive and negative thermal expansion and thermosalient effect in a pentamorphic organometallic martensite. *Nat. Commun.* **5**, 4811 (2014).

7. Collings, I. E., Cairns, A. B., Thompson, A. L., Parker, J. E., Tang, C. C., Tucker, M. G., Catafesta, J., Levelut, C., Haines, J., Dmitriev, V., Pattison, P. & Goodwin, A. L. Homologous Critical Behavior in the Molecular Frameworks $Zn(CN)_2$ and $Cd(imidazolate)_2$. *J. Am. Chem. Soc.* **135**, 7610-7620 (2013).

8. Ogborn, J. M., Collings, I. E., Moggach, S. A., Thompson, A. L. & Goodwin, A. L. Supramolecular mechanics in a metal-organic framework. *Chem. Sci.* **3**, 3011-3017 (2012).

9. Yang, C., Wang, X. P. & Omary, M. A. Crystallographic Observation of Dynamic Gas Adsorption Sites and Thermal Expansion in a Breathable Fluorous Metal-Organic Framework. *Angew. Chem. Int. Ed.* **48**, 2500-2505 (2009).

10. DeVries, L. D., Barron, P. M., Hurley, E. P., Hu, C. H. & Choe, W. "Nanoscale Lattice Fence" in a Metal-Organic Framework: Interplay between Hinged Topology and Highly An isotropic Thermal Response. *J. Am. Chem. Soc.* **133**, 14848-14851 (2011).

11. Grobler, I., Smith, V. J., Bhatt, P. M., Herbert, S. A. & Barbour, L. J. Tunable anisotropic thermal expansion of a porous zinc(II) metal-organic framework. *J. Am. Chem. Soc.* **135**, 6411-6414 (2013).

12. Zhou, H. L., Lin, R. B., He, C. T., Zhang, Y. B., Feng, N. D., Wang, Q., Deng, F., Zhang, J. P. & Chen, X. M. Direct visualization of a guest-triggered crystal deformation based on a flexible ultramicroporous framework. *Nat. Commun.* **4**, 2534 (2013).
